# IL-15 regulates migration, invasion, angiogenesis and genes associated with lipid metabolism and inflammation in prostate cancer

**DOI:** 10.1371/journal.pone.0172786

**Published:** 2017-04-05

**Authors:** Krizia Rohena-Rivera, María M. Sánchez-Vázquez, Diana A. Aponte-Colón, Ingrid S. Forestier-Román, Mario E. Quintero-Aguiló, Magaly Martínez-Ferrer

**Affiliations:** 1 Department of Biochemistry, University of Puerto Rico, School of Medicine, San Juan, Puerto Rico; 2 University of Puerto Rico Comprehensive Cancer Center San Juan, Puerto Rico; 3 Department of Pharmaceutical Sciences, University of Puerto Rico, School of Pharmacy San Juan, Puerto Rico; University of South Alabama Mitchell Cancer Institute, UNITED STATES

## Abstract

Prostate cancer (PCa) is the most commonly diagnosed non-cutaneous cancer. In the United States it is second leading cause of cancer related deaths in men. PCa is often treated via radical prostatectomy (RP). However, 15–30% of the patients develop biochemical recurrence (i.e. increased serum prostate specific antigen (PSA) levels). Interleukin-15 (IL-15) is a secreted cytokine found over expressed in patients with recurrence-free survival after RP. In our study, we aim to determine the role of IL-15 in PCa using *in vitro* and *in vivo* models, and gene expression analysis. PC3 (androgen-independent) and 22RV1 (androgen-dependent) cell lines were treated with IL-15 at 0.0013 ng/mL and 0.1 ng/mL. Tumor growth was evaluated using an orthotopic xenograft model. The anterior prostate lobes of SCID mice were injected with 250,000 22RV1 cells and IL-15 was administered bi-weekly with intraperitoneal (IP) injections during 4 weeks. Tumor tissue was collected for immunohistochemical and gene expression analysis. To study changes in gene expression, we looked at “Tumor Metastasis” and “PI3K pathway” using commercially available PCR arrays. In addition, we employed a microarray approach using the Affymetrix Hugene 2.0 ST array chip followed by analysis with Ingenuity Pathways Analysis (IPA) software. *In vitro* studies showed that IL-15 decreased PCa cell motility at both concentrations. *In vivo* studies showed that IL-15 increased neutrophil infiltration, and the expression of adiponectin, desmin and alpha smooth muscle actin (α-sma) in the tumor tissue. Angiogenesis analysis, using CD31 immunohistochemistry, showed that IL-15 decreased the number of blood vessels. Gene expression analysis identified Cancer, Cell Death, Immune Response and Lipid Metabolism as the major diseases and functions altered in tumors treated with IL-15. This suggests that IL-15 causes inflammation and changes in stroma that can promote decreased tumor cell proliferation.

## Introduction

In the United States of America, prostate cancer (PCa) is the most commonly diagnosed non-cutaneous cancer, and the second leading cause of death in male patients [1]. PCa is often treated with radical prostatectomy (RP). Even though it can provide positive results, 15–30% of the patients will suffer from biochemical recurrence (BCR) or elevated blood levels of prostate specific antigen (PSA) [2,3]. BCR often develops asymptomatically within 10 years of treatment and can occur due to cells that have metastasized to other areas [4,5]. Although BCR can be identified with elevated PSA levels, there are currently no reliable predictive biomarkers in the clinic that can be used to asses biochemical recurrence risk [5,6]. The available biomarkers and (or) clinical information are insufficient to predict recurrence and metastasis [7]. The early detection of potential metastatic or recurrent PCa through the use of novel biomarkers can lead to proactive use of adjuvant therapeutic options and better patient outcomes.

Given that inflammation plays a significant role in cancer progression, the expression of inflammatory mediators, such as cytokines, is important in PCa. Studies have found that the expression of cytokines and their receptors can vary with stage and aggressiveness, and can fluctuate during treatment [8–10]. Thus, cytokines have been implicated as potential biomarkers for predicting PCa progression and recurrence.

IL-15, a 15 kDa protein and member of the 4-alpha-helix bundle family of cytokines, is a pro inflammatory cytokine with very similar functions to Interleukin 2 (IL-2). It binds to the specific receptor IL-15Rα and activates the Jak1/Jak3/Stat5 signaling pathway [11]. IL-15 plays an important role in the maturation and proliferation of T, B and NK cells. More specifically, IL-15 increases the cytotoxicity of CD8+ T cells and is essential for NK cell activation [12]. Given its ability to attract CD8+ T cells and NK cells towards the tumor site, IL-15 has been identified as an anti-tumor cytokine in several models, including: neuroblastoma, breast and colorectal cancer [13–16]. In the context of PCa, IL-15 has been associated with recurrence-free survival after RP [17]. This suggests that IL-15 expression in the microenvironment may provide a benefit for PCa patients. Our study seeks to provide insight to the considerable gaps in the existing knowledge that connects the biologic role of IL-15 and positive outcomes in PCa patients.

In addition to inflammation, differences in gene expression patterns and genome instability are associated with cancer progression [18,19]. In PCa, one of the most studied gene mutations is the loss of phosphatatse and tensin homolog (PTEN). PTEN, a tumor suppressor gene, encodes a tyrosine phosphatase that modulates cell cycle progression [20]. With mutation rates of up to 60% in localized cancer, PTEN deletion is one of the most common mutations in PCa and it is often associated with poor prognosis [21–23]. In addition to PTEN loss, other gene expression patterns have been associated with high Gleason Score and PCa relapse [24]. Thus, gene expression patterns can be used to stratify patients and to understand the mechanisms that promote PCa progression. Therefore, in this project we aim to identify, gene expression patterns affected by IL-15 in PCa.

In this study, we evaluated the role of IL-15 in migration, invasion, proliferation, tumor growth, and angiogenesis using *in vitro* and *in vivo* models of PCa. In addition we focused on gene expression changes that could predict tumor progression in the long term. We show that IL-15 decreased invasion and migration of PCa cells without affecting growth *in vitro*. We also demonstrate that IL-15 causes an increase in neutrophil infiltration in the tumor tissue. Moreover, adiponectin, desmin, and alpha smooth muscle actin (α-sma) expression was increased with IL-15 treatment *in vivo*. These findings suggest that IL-15 causes inflammation and changes in stroma that can promote a decrease in tumor cell proliferation. To investigate the effect of IL-15 in angiogenesis, we analyzed CD31 expression. We show that IL-15 decreases the number of blood vessels, suggesting that IL-15 decreases angiogenic potential *in vivo*. In summary, we show that IL-15 reduces cell migration and invasion *in vitro*. In addition, IL-15 reduces proliferation *in vivo* (as shown by pH3 expression), reduces angiogenic potential (as shown by CD31) and modifies the stroma (as shown by desmin and α-sma expression). For gene expression studies, we first looked at two specific pathways “Tumor Metastasis” and “PI3K pathway” using commercially available PCR arrays. Afterwards, we used a microarray approach with the Affymetrix Hugene 2.0 ST array chip. Data obtained from the microarray chip was analyzed with Ingenuity Pathway Analysis (IPA) software. We identified four major networks: Cancer, Cell Death, Immune Response and Lipid Metabolism. These data suggest that IL-15 treatment could inhibit PCa progression by promoting an immune response that can cause cell death. Thus, our study provides evidence that IL-15 regulates migration, invasion, angiogenesis and genes associated with lipid metabolism and inflammation in PCa.

## Materials and methods

The Medical Sciences Campus Institutional Animal Care and Use Committee (IACUC) approved protocol number A8700110 to perform this project.

### Cell culture

PC3 (androgen independent) and 22RV1 (androgen dependent) PCa cell lines were obtained from the American Type Culture Collection (ATCC) (Manassas, VA, USA). Cells were cultured in complete RPMI-1640 medium (Hyclone, Waltham, MA, USA) with 10% fetal bovine serum (FBS) (Hyclone, Waltham, MA, USA) and penicillin-streptomycin complex (1,000 units/mL) (Gibco, Life Technologies, Carlsbad, CA, USA) at 37°C and 5% CO_2_ in a humidified incubator.

### Scratch wound healing assay

In 12-well tissue culture plates, PC3 cells (2 X 10^5^ cells/mL) were maintained until 95% confluent. After 24 hours of serum starvation, a wound was made in the cell monolayer using a 200 μL pipette tip. Cells were washed using PBS and treated with complete RPMI medium containing IL-15 (Genway, San Diego, CA, USA) (0.0013 ng/mL and 0.1 ng/mL), or control (PBS). Images at a 4x magnification were obtained with a Nikon Eclipse TS100 microscope (Nikon, Tokyo, Japan) at 0, 12 and 24 hours of treatment. Within each wound, we analyzed 10 distance measurements using Image Pro Plus Software. The wound closure differences were normalized and compared to the control using Student’s T-test at a 95% confidence interval. Experiments were performed in triplicate.

### Invasion assay

After 24 hours of serum starvation, 22RV1 and PC3 cells were seeded at a density of 4 X 10^4^ cells/mL in 24-well 8.0 μm pore transwell chambers (Corning, Corning, NY, USA) coated with laminin/entactin (Becton Dickinson, Franklin Lakes, NJ, USA). The reservoir well had complete RPMI 1640 medium with IL-15 for a final concentration of 0.0013 ng/mL or 0.1 ng/mL. Under culturing conditions (37°C and 5% CO_2_), the cells were allowed to invade during 24 hours. With a sterile cotton swab and PBS, we removed the non-invasive cells from the top chamber. Invasive cells were fixed for 30 minutes, with 10% formalin (Thermo Scientific Waltham, MA, USA) and stained over night with hematoxylin (American Master Tech, Lodi, CA, USA). The membranes were washed with water and mounted on slides. Photographs at a 4x magnification were captured with a Nikon Eclipse TS100 microscope (Nikon, Tokyo, Japan). The number of invasive cells was counted using Image Pro Plus Software. Results were analyzed using the Student’s T-test at a 95% confidence interval. All experiments were performed in triplicate.

### Orthotopic mouse model

Male ICR-SCID mice (IcrTac:IcrCrl-SCID) (Taconic, Germantown, NY, USA) (7–8 weeks old) were kept in a pathogen-free environment under the Institutional Animal Care and Use Committee regulations at The University of Puerto Rico Medical Sciences Campus animal facility (protocol #A8700110). Mice received food and water ad libitum with a 12-hour light cycle. To develop 2 prostate tumors per mouse, we performed an orthotopic xenograft model in which 22RV1 (250,000 cells) were injected in the anterior prostate lobes of ICR-SCID mice. To reduce leakage in the peritoneal cavity during surgery, cell suspensions in PBS were placed in 30 μL of collagen I (Becton Dickinson, Franklin Lakes, NJ, USA) and allowed to partially solidify. IL-15 (0.0013 ng/mL) or vehicle (Saline) was administered bi-weekly with intraperitoneal injections during 4 weeks. In total, the control group had 13 mice and the IL-15 group had 10 mice. Each mouse yielded 2 tumors. Tumor volume was determined using caliper measurements. Results were analyzed using the Student’s T-test at a 95% confidence interval. To evaluate metastasis, we collected lung, liver, and spleen organs. We performed gross examination for visible nodules and hematoxylin eosin staining to certify any metastatic lesions.

### Tissue collection and processing

Tumor samples (n_control_ = 26 tumors, n_IL-15_ = 20 tumors) were divided in two sections. One was frozen in dry ice and stored at -80°C, while the other was fixed in 10% buffered formalin. The frozen tissue section was used to isolate RNA for gene expression assays. The fixed tissue was processed and paraffin-embedded.

### Hematoxylin-eosin staining

For histological examination, 5 μm sections of formalin-fixed paraffin-embedded (FFPE) tissue were deparaffinized in xylene and hydrated using serial descending concentrations of alcohol. Staining with hematoxylin was followed by stain differentiation with 1% v/v acid alcohol (80% ethanol, 19% deionized water, 1% HCl), 0.3% v/v ammonia water (0.3% NH_4_OH in de-ionized H_2_0) and washing with 70% ethanol. After eosin staining (0.05% Eosin Y in 70% Ethanol-0.005% acetic acid) the tissue sections were dehydrated with increasing serial dilutions of ethanol and xylene. Slides were mounted using permount mounting medium. n = 5 representative tumors per group.

### Immunohistochemistry and immunofluorescence

FFPE tumor samples were dewaxed in xylene and rehydrated in descending concentrations of alcohol. Antigen retrieval was performed using heat and a citrate-based Antigen Unmasking Solution (1:100 dilution) (Vector Laboratories, Burlingame, Ca, USA). Endogenous peroxidase was quenched with 3% v/v H_2_O_2_. The primary antibodies used were: phospho-histone 3 (pH3) (1:1000 dilution) (Abcam, Cambridge; MA, USA), CD31 (1:50 dilution) (Abcam, Cambridge; MA, USA), desmin (1:1000 dilution) (Santa Cruz Biotechnology, Santa Cruz, CA, USA), alpha-smooth muscle actin (a-sma) (1:25 dilution) (Thermo Scientific Waltham, MA, USA), adiponectin (1:100 dilution) (Abcam, Cambridge; MA, USA), and neutrophil elastase (1:1000 dilution) (Abcam, Cambridge; MA, USA). All immunohistochemistry was detected using Dako Envision system-HRP (DAB) (anti-rabbit) (Dako; Glostrup, Denmark) or Dako LSAB System-HRP (DAB) (anti-mouse) (Dako; Glostrup, Denmark) according to the manufacturer’s instructions. Hematoxylin was used as a counterstain. For immunofluorescence, the secondary antibody used was Alexa-Fluor 594 (anti-rabbit) 1:2000 (Molecular Probes, Life Technologies, Carlsbad, CA, USA) and nuclei were stained with DAPI 1:5000 (Santa Cruz Biotechnology, Santa Cruz, CA, USA). To quantify pH3, desmin, a-sma and adiponectin, a subjective scale from 1–4 was used. Here, we gave a score of one (1) if 25% or less of the tumor cells were stained, a score of two (2) if 26% to 50% of the tumor cells were stained, a score of three (3) if 51% to 75% of the tumor cells were stained, and a score of four (4) if more than 75% of the tumor cells were stained. Score was given in a blind manner. N = 5 representative tumors per group. To quantify CD31, a set of 3 random fields was chosen per slide and the total number of blood vessels was counted. To quantify neutrophil elastase, 3 random fields were chosen per slide and the total number of positive cells was counted. Statistical analysis was done using the Student’s T-test at a 95% confidence interval. n = 5 representative tumors per group.

### PCR array analysis

To evaluate changes in gene expression the Qiagen RT^2^ PCR arrays (human tumor metastasis and PI3K pathway) were used (Qiagen Inc., Valencia; CA, USA). RNA (2 μg per array) was reverse transcribed using the RT^2^ First Strand Kit including DNA elimination procedure (Qiagen Inc., Valencia; CA, USA). Results were analyzed using the MS Excel based tool provided by Qiagen (PCR Array Analysis V4, available for download at https://www.qiagen.com/us/resources/resourcedetail?id=d8d1813e-e5ba-4d29-8fdf-07a3f4227e0a&lang=en). We used was a standard two-step SYBR green amplification cycle (95°C for 15 seconds and 60°C for 1 minute). n = 3 tumors per group, randomly chosen.

### Microarray analysis

Affymetrix Hugene 2.0 chip based transcript profiling was performed at the RCMI Center for Genomics in Health disparities and Rare Diseases (University of Puerto Rico, Medical Sciences Campus). Following quality control, the RNA was prepared for microarray analysis using the standard Affymetrix protocol (Affymetrix Inc, Santa Clara, CA). Total RNA (100 ng) was converted to cDNA and amplified using T7 oligo dT and the GeneChip® WT cDNA Synthesis Kit, the GeneChip® WT cDNA Amplification Kit, and the GeneChip® Sample Cleanup Module as described in the GeneChip® Whole Transcript (WT) Sense Target Labeling Assay Manual Addendum. All quality control steps were followed to ensure that the RNA was adequate for later use in the first strand cDNA synthesis (where 10 μg are required), that the yield of cDNA was ≥ 5.5 μg of Single-Stranded DNA and that the fragmentation step worked properly by size analysis with the RNA 6000 Nano LabChip Kit in the Agilent Bioanalyzer. A gel-shift analysis of the WT (Whole Transcript) was done to assess the labeling efficiency of the fragmented cDNAs. The image data was normalized using the Expression Console software provided by Affymetrix. The mode of analysis used was Gene Level RMA sketch (S7–S10 Files). The QC metrics were verified to certify that the hybridization was performed correctly (S11 File). The intensity boxplot was observed to ensure that all samples had uniform intensity values to proceed with the analysis (S5 Fig). The signal distribution among arrays was observed to certify that all arrays exhibit a uniform signal distribution S6 Fig). Gene expression values and clustering was done using the Transcriptome Analysis Console also provided by Affymetrix. The settings used to identify differences in expression were, a fold change higher that 2 or lower than -2 and a p value lower than 0.05. Identification of gene expression patterns was done with IPA software. The settings for this final analysis were a fold change higher than 1.5 or lower than -1.5 and a p value lower than 0.05. To identify the affected functions and networks we used the “diseases and functions” sections of the IPA software. We selected the complete array of functions and selected the top four representative functions based on score. Data was documented according to the MIAME guidelines [25]. To see detailed information of this procedure look at the supplementary information (S1 File)

### Real time PCR validation

To validate PCR array and microarray results, quantitative Real time PCR (qRT-PCR) was performed under standard conditions using the Step One Plus Real-time PCR System (Applied Biosystems, Carlsbad; CA, USA). For each gene of interest (GOI), the primers where designed with the Integrated DNA Technologies (IDT) Primer Quest tool. To ensure specificity, we performed a BLAST for each sequence. Real-time PCR was performed in 10 μL reactions using SYBR super mix (Bio-Rad, Hercules, CA, USA). Depending on the GOI, the cycle used was, 95°C for 15 seconds and 62°C for 1 minute or 95°C for 15 seconds and 56°C for 1 minute. PCR efficiency was examined and the melting curve data was collected for PCR specificity. The housekeeping gene used was GAPDH. Quantification was done using the ΔΔC_t_ method. No PCR product was detected in control samples in which the template was omitted. Statistical analysis was done with a Mann-Whitney U test at a 95% confidence interval. (n = 5 tumors per group).

### Statistical analysis

All experimental procedures were performed in triplicate. *In vitro* and immunostaining procedures were analyzed using a student T-test. Real time PCR results were analyzed using a Mann Whitney U test. All statistical procedures were performed with the GraphPad Prism Software (GraphPad Software, Inc CA, USA). Significance for all assays was accepted at a 95% confidence interval (P < 0.05).

## Results

### IL-15 decreases prostate cancer cell motility and invasion

**Fig 1:**
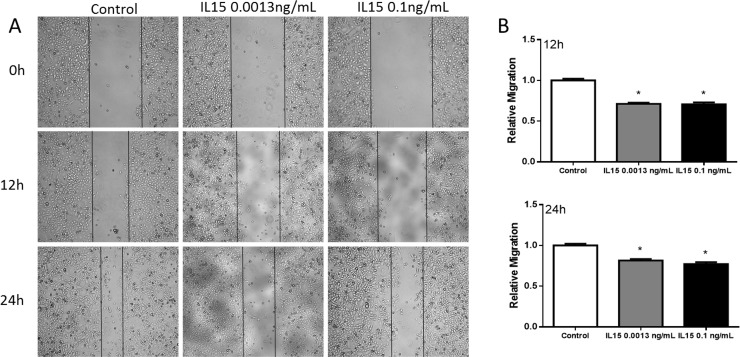
IL-15 decreases PC3 cell migration *in vitro*. (A) Representative 4x magnification images at 0, 12, and 24hours (top to bottom). (B) Statistical analysis shows that IL-15 treatment causes a significant decrease in cell migration at 12 hours (top) and 24 hours (bottom). Mean + SEM (*P<0.05).

To determine the effect of IL-15 in cell motility, we performed a wound-healing assay. PC3 cells were cultured to confluence. The monolayer was wounded and allowed to migrate for 12 and 24 hours with PBS (control), IL-15 at 0.0013 ng/mL and IL-15 at 0.1 ng/mL. After 12 hours, IL-15 treatment at 0.0013 ng/mL and 0.1 ng/mL reduced the migration of PC3 cells by 30% when compared to control (P<0.05) (Fig 1). At 24 hours, the migration of PC3 cells was reduced by 20% in cells treated with IL-15 at 0.0013 ng/mL and IL-15 at 0.1 ng/mL when compared to control. 22RV1 cells were not used for the wound-healing assay because these cells do not grow in a confluent monolayer.

**Fig 2:**
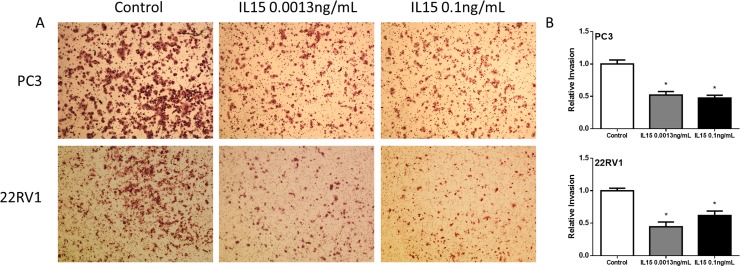
IL-15 decreases PC3 and 22RV1 cell invasion *in vitro*. (A) Representative 10x magnification images of invasive cells at 24h. PC3 (Top) 22RV1 (Bottom) (B) Statistical analysis shows that IL-15 treatment causes a significant decrease in cell invasion. PC3 (Top) 22RV1 (Bottom). Mean + SEM (*P<0.05).

To study cell invasion, we performed a boyden chamber assay during 24 hours. We found that IL-15 at 0.0013 ng/mL and 0.1 ng/mL reduced the invasion of both PC3 and 22RV1 cells by 50% (P<0.05) (Fig 2). Additionally, we studied cell growth using an MTS-based assay. We found no significant differences for either PC3 or 22RV1 cell lines (Data not shown). These results show that IL-15 inhibits cancer cell motility and invasion in PCa cells while cell growth remained unaffected.

### IL-15 increases tumor volume without promoting cancer cell proliferation or angiogenesis

**Fig 3:**
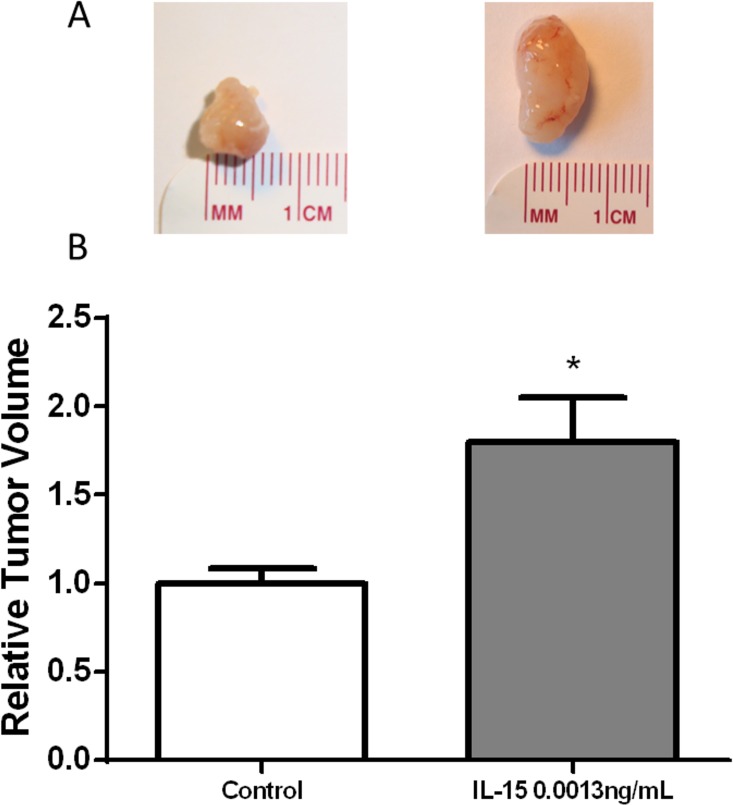
IL-15 increases tumor volume. (A) Representative photographs of murine tumor tissue treated with IL-15 (0.0013 ng/mL) and control. (B) Statistical analysis shows that IL-15 increased tumor volume at 0.0013ng/mL. N_control_ = 26, N_IL-15_ = 20. Mean + SEM (*P<0.05).

The effect of IL-15 in tumor growth was studied using an orthotopic model in which the anterior prostate lobes of SCID mice were injected with 250,000 22RV1 cells in a collagen-1/PBS suspension. Chemokine treatment proceeded during four weeks with bi-weekly intraperitoneal injections of saline solution for the control group and IL-15 0.0013 ng/mL solution for the treatment group. Mice treated with IL-15 intraperitoneal injections (0.001 ng/mL) developed significantly larger tumors when compared to the control (P<0.05) (Fig 3).

**Fig 4:**
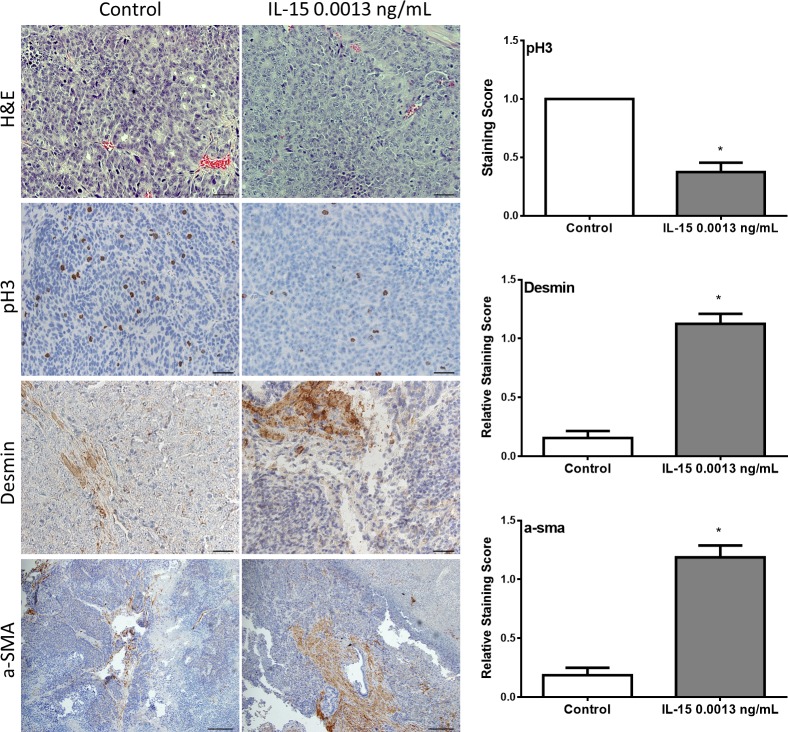
IL-15 treatment affects expression of pH3, desmin and a-sma *in vivo*. Tumor tissue was evaluated pathologically and immunohistochemically. Pathological analysis was done with hematoxylin-eosin staining (top pane) and immunohistochemistry was done to evaluate the expression of phosho-histone 3 (pH3), desmin, and alpha smooth muscle actin (a-sma), (top to bottom). IL-15 treatment decreased the expression of pH3, and increased the expression of desmin and a-sma. n = 10 tumors per group. Scale bar (H&E, pH3, and desmin) = 20 μm (40x), Scale bar (a-sma) = 50 μm (20x) Mean + SEM (*P<0.05)

Pathological, histological and immunohistochemical analysis of collected tumor tissue was used to study the effect of IL-15 in tumor biology. Slides were examined by a pathologist at low, medium and high power under a compound light microscope. Tumor assessment was made as described by Lsaacs and Hukku [26]. Tumors were classified in four categories by degree of differentiation: well-differentiated, moderately-differentiated, poorly-differentiated, and anaplastic. Well-differentiated tumors are characterized by the presence of glandular structures, lumen, basement membrane, and stroma. Moderately-differentiated tumors are characterized by smaller glandular structures with the lumen obstructed by tumor cells. However, the basement membrane and stroma remained intact. Tumors classified as poorly-differentiated have absence of glandular structures, basement membrane, and do not show a consistent relationship between tumor cells and stroma. Individual tumor cells, however, still show a normal nucleus to cytoplasm ratio. Tumors classified as anaplastic lack appearance of tissue organization and individual tumor cells show irregular nucleus size and abnormal nucleus to cytoplasm ratio. All tumor samples, regardless of the treatment, were classified as histologically anaplastic showing no significant differences among treatments (Fig 4). To determine if IL-15 increased tumor volume by increasing cell proliferation *in vivo*, we measured the expression of phospho-histone 3 (pH3) by immunohistochemistry (Fig 4). Tumors developed in mice treated with IL-15 showed a significant decrease in pH3 expression (Fig 4). To study changes in stroma, we measured the expression of desmin and alpha smooth muscle actin (a-sma) by immunohistochemistry (Fig 4). Our results showed that tumors developed in mice treated with IL-15 at 0.0013 ng/mL had increased expression of desmin and a-sma (Fig 4).

**Fig 5:**
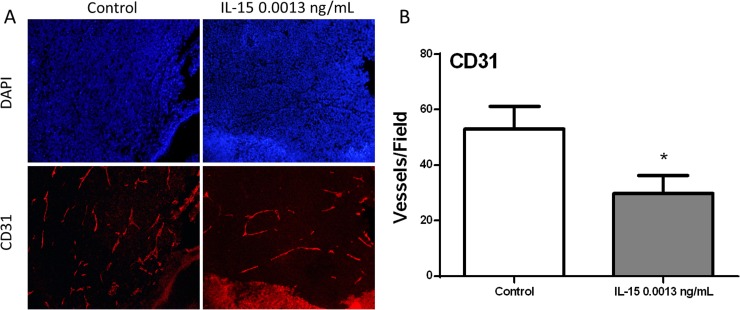
IL-15 decreases angiogenesis *in vivo*. To evaluate angiogenesis, we performed Immunofluorescence of CD31 in tumor tissue. (A) Representative 20x magnification immunofluorescence images of 22RV1 tumors, Control and IL-15 0.001ng/mL. Nuclei are stained with DAPI (blue) and blood vessels are stained with CD31 (red). (B) Statistical analysis shows that blood vessels were significantly decreased with IL-15 treatment. Mean + SEM (*P<0.05).

During gross examination, we observed that tumors developed in mice treated with IL-15 at 0.0013 ng/mL were less vascular than control tumors. Therefore, we evaluated angiogenesis measuring CD31 expression by immunofluorescence (Fig 5A). Our results showed that IL-15 at 0.0013 ng/mL significantly decreased the number of blood vessels in tumor tissue (P<0.05) (Fig 5B). These results show that IL-15 increased tumor volume, however, the number of actively proliferating cells and the amount of blood vessels were significantly decreased. Additionally, we observed changes in stroma with the increased expression of desmin and a-sma. These data suggest that increase in tumor volume may be caused by factors unrelated to cancer progression.

### IL-15 increases lipid deposition and inflammation, which contributes to an increase in tumor volume

**Fig 6:**
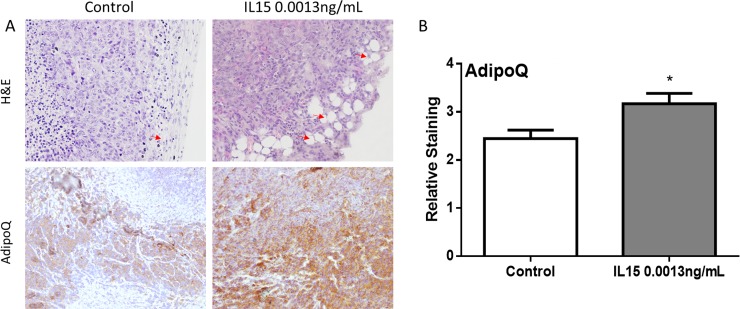
IL-15 increases lipid deposition and metabolism *in vivo*. (A) Representative 40x magnification images of 22RV1 tumors, H&E (Top panel) shows increased number of lipid droplets in IL-15 tumors (Red arrows), AdipoQ (Bottom Panel) shows increased expression of adiponectin in IL-15 tumors. (B) Statistical analysis shows that adiponectin is significantly increased with IL-15 treatment. Mean + SEM (*P<0.05).

**Fig 7:**
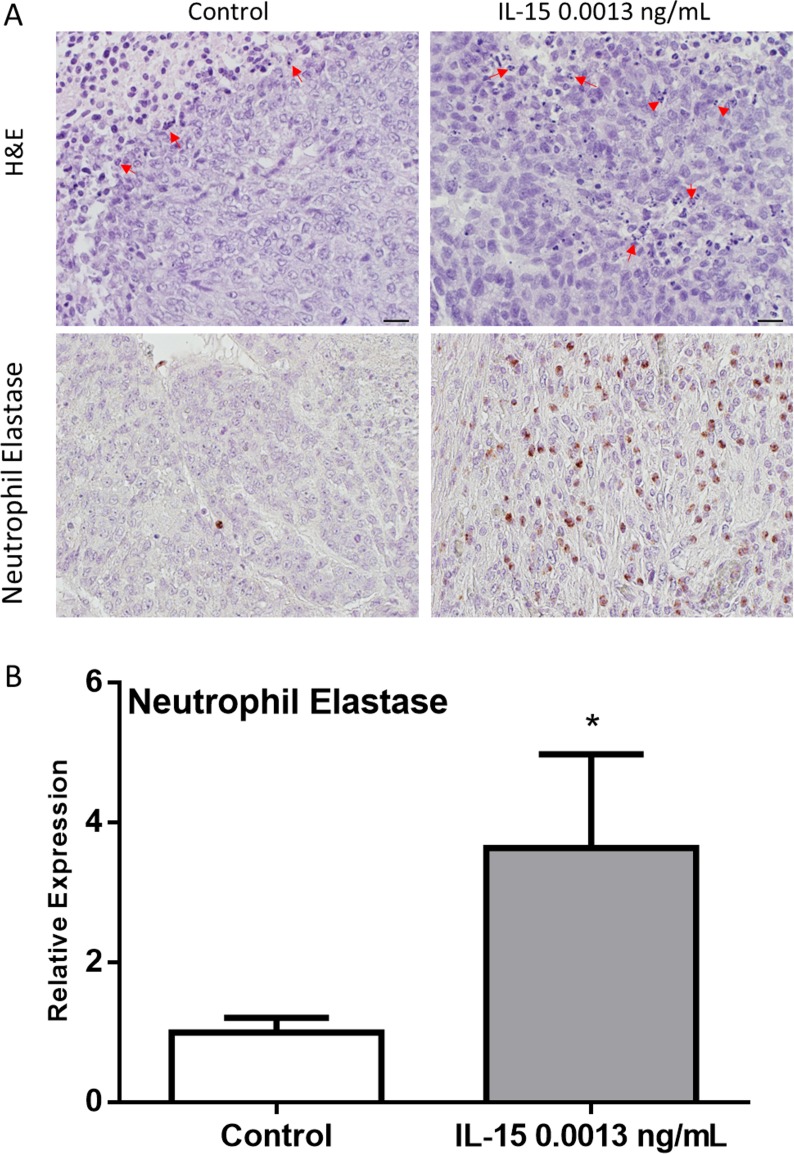
IL-15 increases neutrophil invasion and degranulation *in vivo*. (A) Representative images of 22RV1 tumors: H&E (Top panel) shows increased invading neutrophils (60x magnification) (red arrows) in IL-15; neutrophil elastase (Bottom Panel) shows increased expression of Neutrophil elastase in IL-15 tumors (40x magnification). (B) Statistical analysis shows that neutrophil elastase was significantly increased with IL-15 treatment. Scale bar = 10 μm, Mean + SEM (*P<0.05).

After observing decreased cancer cell proliferation and angiogenesis in mice treated with IL-15, we hypothesized that increased tumor growth could be attributed to other factors. During gross examination, tumors generated under IL-15 treatment appeared fattier than control tumors. As a result, we decided to look for lipid deposition. A pathologist examined the slides and identified adipocytes as empty spaces of lipid droplets. Tumors generated in mice treated with IL-15 at 0.0013 ng/mL had increased number of adipocytes infiltrating the tumor cells (Fig 6A, top panel). To determine if IL-15 increases lipid mobilization and metabolism we verified adiponectin (adipoq) expression using immunohistochemistry. Our results show that IL-15 significantly increases the expression of adiponectin *in vivo* (Fig 6B, bottom panel). Additionally, given that IL-15 is a pro-inflammatory cytokine, we examined the tumors for signs of inflammation. Slides were examined for the presence of neutrophils which appear as multinucleated cells. Upon examination, we observed that IL-15 treatment at 0.0013 ng/mL increases the number of neutrophils. In addition, we observed that the neutrophils were infiltrating the tumor (Fig 7A). Furthermore, to examine the expression of neutrophil elastase we performed IHC. Our results confirm that IL-15 significantly increases the expression of neutrophil elastase *in vivo* (Fig 7B). These results suggest that IL-15 increases tumor volume by promoting lipid deposition, lipid metabolism, and inflammation.

### IL-15 treatment leads to deregulation of genes involved in metastasis and the PI3K pathway

After observing decreased cell motility and angiogenesis as a result of IL-15 treatment, we decided to assess the changes in gene expression using a PCR array approach. We focused on tumor metastasis and the PI3K pathway. Results from these arrays suggest that IL-15 affected the expression of MMPs and TIMPs associated with tumor metastasis (Table 1). In addition, IL-15 also modulates several genes associated with the PI3K pathway (Table 2). We confirmed these changes in gene expression through real time PCR. The primer sequences used for qRT-PCR are listed in Table 3.

**Table 1:** PCR array: Genes associated with tumor metastasis differentially expressed by IL-15 *in vivo*.

**Gene Symbol**	**Gene Accession Number**	**Description**	**Fold Change**
MMP2	NM_004530	Matrix metallopeptidase 2 (gelatinase A, 72kDa gelatinase, 72kDa type IV collagenase)	-1.52
MMP7	NM_002423	Matrix metallopeptidase 7 (matrilysin, uterine)	-1.50
MMP9	NM_004994	Matrix metallopeptidase 9 (gelatinase B, 92kDa gelatinase, 92kDa type IV collagenase)	1.99
MMP10	NM_002425	Matrix metallopeptidase 10 (stromelysin 2)	-1.51
MMP11	NM_005940	Matrix metallopeptidase 11 (stromelysin 3)	-1.19x10^9^
TIMP2	NM_003255	TIMP metallopeptidase inhibitor 2	-1.42
TIMP3	NM_000362	TIMP metallopeptidase inhibitor 3	21.06

**Table 2:** PCR array: Genes associated with the PI3K pathway differentially expressed by IL-15 *in vivo*.

**Gene Symbol**	**Gene Accession Number**	**Description**	**Fold Change**	**p value**
AKT3	NM_005465	V-akt murine thymoma viral oncogene homolog 3 (protein kinase B, gamma)	2.51	0.3835
BTK	NM_000061.2	Bruton agammaglobulinemia tyrosine kinase	4.95	0.3484
CCND1	NM_053056.2	Cyclin D1	-3.23	0.0577
CD14	NM_000591.3	CD14 molecule	3.75	0.3587
FASLG	NM_000639.2	Fas ligand (TNF superfamily, member 6)	5.06	0.3478
FOXO3	NM_001455	Forkhead box O3	-2.02	0.3089
HSPB1	NM_001540.3	Heat shock 27kDa protein 1	-3.15	0.1417
IGF1	NM_000618	Insulin-like growth factor 1 (somatomedin C)	6.26	0.1102
IGF1R	NM_000875	Insulin-like growth factor 1 receptor	-3.19	0.1811
ILK	NM_004517.3	Integrin-linked kinase	-2.00	0.0573
IRS1	NM_005544.2	Insulin receptor substrate 1	-2.06	0.2651
MAP2K1	NM_002755.3	Mitogen-activated protein kinase kinase 1	-2.00	0.2001
PDGFRA	NM_006206.4	Platelet-derived growth factor receptor, alpha polypeptide	5.06	0.3478
PIK3CG	NM_002649	Phosphoinositide-3-kinase, catalytic, gamma polypeptide	5.06	0.3478
PIK3R1	NM_181504.3	Phosphoinositide-3-kinase, regulatory subunit 1 (alpha)	-2.01	0.0344
PIK3R2	NM_005027.3	Phosphoinositide-3-kinase, regulatory subunit 2 (beta)	-2.48	0.0404
PTEN	NM_000314	Phosphatase and tensin homolog	3.11	0.1919
TLR4	NM_138554.4	Toll-like receptor 4	4.81	0.3490

**Table 3:** Primer sequences used for qRTPCR confirmation of IL-15 PCR array identified genes.

**Gene Symbol**	**Gene Accession Number**	**Forward Primer**	**Reverse Primer**
AKT3	NM_005465	CTCTGGAGTAAACTGGCAAGATG	GATTGCTGACATTTTTCAGGTGG
BTK	NM_000061.2	GAAAGGTTCCCTTATCCCTTCC	GAATCCACCGCTTCCTTAGTT
CCND1	NM_053056.2	GGTTCAACCCACAGCTACTT	CAGCGCTATTTCCTACACCTATT
CD14	NM_000591.3	CTTGTGAGCTGGACGATGAA	TGCAGACACACACTGGAAG
FASLG	NM_000639.2	GTAGCTCCTCAACTCACCTAATG	TTCATGCTTCTCCCTCTTCAC
FOXO3	NM_001455	AGAGCTGAGACCAGGGTAAA	GACAGGCTTCACTACCAGATTC
HSPB1	NM_001540.3	CTCAAACACCGCCTGCTAAA	TCTGGACGTCTGCTCAGAAA
IGF1	NM_000618	TGGTCCTGGAGTTGGTAGAT	TTGAGAGGCAGGGACTAAGA
IGF1R	NM_000875	TTCTCCCTTTCTCTCTCCTCTC	GACAGCCACTTCCTCAAACT
ILK	NM_004517.3	CCCACGACATGCACTCAATA	GACCAGGACATTGGAAAGAGAA
IRS1	NM_005544.2	CTTTCCACAGCTCACCTTCT	CCAGGTCCATCTTCATGTACTC
MAP2K1	NM_002755.3	GTGGGAGAACTGAAGGATGAC	TTGTGGGAGACCTTGAACAC
PDGFRA	NM_006206.4	CGGAATAACATCGGAGGAGAAG	TGAAAGCTGGCAGAGGATTAG
PIK3CG	NM_002649	TGGATATGAAGGGAGCCCCA	CATGCCCTATGCGACCTGAT
PIK3R1	NM_181504.3	GCTTTGCCGAGCCCTATAA	ACATTGAGGGAGTCGTTGTG
PIK3R2	NM_005027.3	GTGGACCTCATCAATCACTACC	ATCTGGTCCTGCTGGTATTTG
PTEN	NM_000314	CCCACCACAGCTAGAACTTATC	TCGTCCCTTTCCAGCTTTAC
TLR4	NM_138554.4	GATGAGGACTGGGTAAGGAATG	GGCCACACCGGGAATAAA
MMP2	NM_004530	AGAGAACCTCAGGGAGAGTAAG	CCTCGAACAGATGCCACAATA
MMP7	NM_002423	CACTGTTCCTCCACTCCATTTA	GACATCTACCCACTGCAAGTATAG
MMP9	NM_004994	GGGCTTAGATCATTCCTCAGTG	GCCATTCACGTCGTCCTTAT
MMP10	NM_002425	GGCCCTCTCTTCCATCATATTT	CCTGCTTGTACCTCATTTCCT
MMP11	NM_005940	AAGACGGACCTCACCTACA	GTCACATCGCTCCATACCTTTA
TIMP2	NM_003255	AGGGCCTGAGAAGGATATAGAG	GGCCTTTCCTGCAATGAGATA
TIMP3	NM_000362	CATCTCTTCTGCCTCCCAATC	AGTCTCCCAGTATTACCTACCC

**Fig 8:**
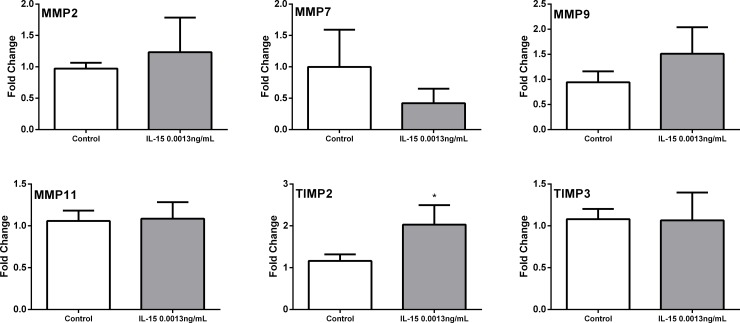
qRTPCR analysis of differentially expressed genes in murine tumors treated with IL-15: Tumor Metastasis PCR array. Genes were obtained from the Tumor metastasis PCR array. Expression of: Matrix metallopeptidase 2 (MMP2), Matrix metallopeptidase 7 (MMP7), Matrix metallopeptidase 9 (MMP9), Matrix metallopeptidase 11 (MMP11), Tissue inhibitor of metallopeptidase type 2 (TIMP2) and Tissue inhibitor of metallopeptidase type 3 (TIMP3). Fold change was calculated with the ddCT method. N = 5 representative tumor samples per treatment. Mean+ SEM. *p<0.05. Experiments performed in triplicate.

**Fig 9:**
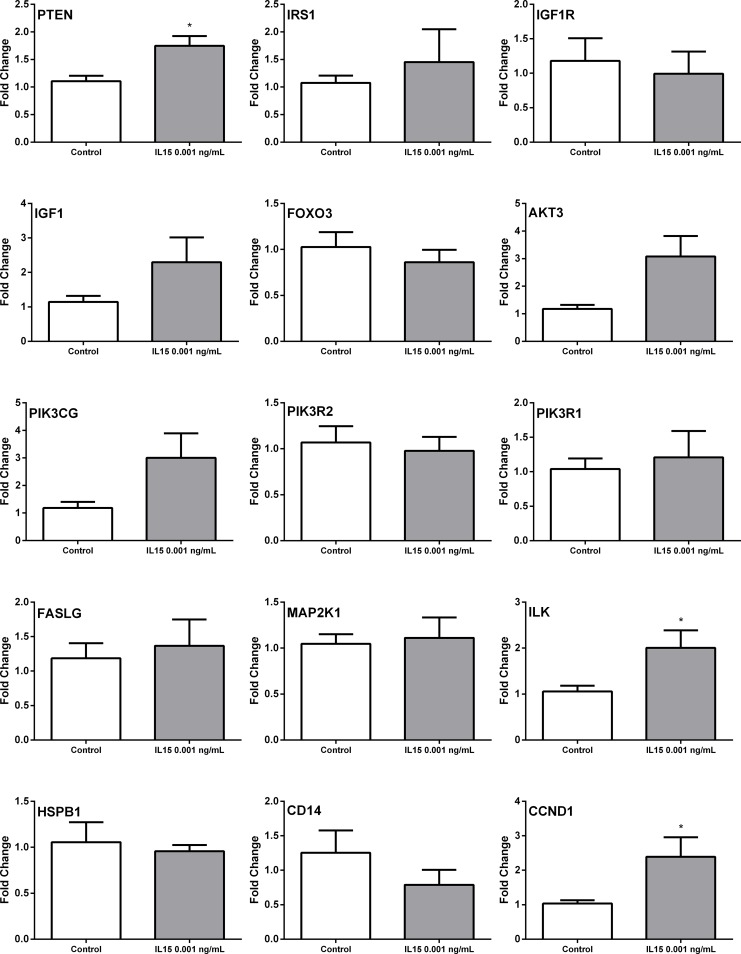
qRTPCR analysis of differentially expressed genes in murine tumors treated with IL-15: PI3K PCR array. Genes were obtained from the PI3K pathway PCR array Real time PCR results for: Phosphatase and tensin homolog (PTEN), Insulin receptor substrate 1 (IRS1), Insulin-like growth factor 1 receptor (IGF1R), Insulin-like growth factor 1 (IGF1), Forkhead box O3 (FOXO3), V-akt murine thymoma viral oncogene homolog 3 (AKT3), Phosphoinositide-3-kinase, catalytic, gamma polypeptide (PIK3CG), Phosphoinositide-3-kinase, regulatory subunit 2 (beta) (PIK3R2), Phosphoinositide-3-kinase, regulatory subunit 1 (alpha) (PIK3R1), Fas ligand (FASLG), Mitogen-activated protein kinase kinase 1 (MAP2K1), Integrin-linked kinase (ILK), Heat shock 27kDa protein 1 (HSPBP1), CD14 molecule (CD14), and Cyclin D1 (CCND1). Fold change calculated with the ddCT method. N = 5 representative tumor samples per treatment. Mean+ SEM. *p<0.05. Experiments performed in triplicate.

Results from real time PCR confirmation show that IL-15 caused no significant changes in the expression of matrix metallopeptidase 2 (MMP2) and matrix metallopeptidase 11 (MMP11), and TIMP metallopeptidase inhibitor 3 (TIMP3). Although changes in matrix metallopeptidase 9 (MMP9) and matrix metallopeptidase 7 (MMP7) were not statistically significant, the expression was different. Nevertheless, one natural inhibitor of these proteases, TIMP metallopeptidase inhibitor 2 (TIMP2) was increased. (Fig 8). In addition, IL-15 increased the expression of phosphatase and tensin homolog (PTEN), insulin receptor substrate 1 (IRS1), insulin-like growth factor 1 (IGF1), phosphoinositide-3-kinase, catalytic, gamma polypeptide (PIK3CG), Fas ligand (FASLG), Integrin-linked kinase (ILK), CD14 molecule (CD14) and Cyclin D1 (CCND1) (Fig 9). These results suggest that IL-15 cause changes in the stroma as shown by the differences in expression of MMPs and TIMPs. Additionally, the over expression of PTEN suggest that IL-15 could increase the expression of tumor suppression genes.

### IL-15 alters the expression of genes related to cancer, cell death immune response, and lipid metabolism

**Fig 10:**
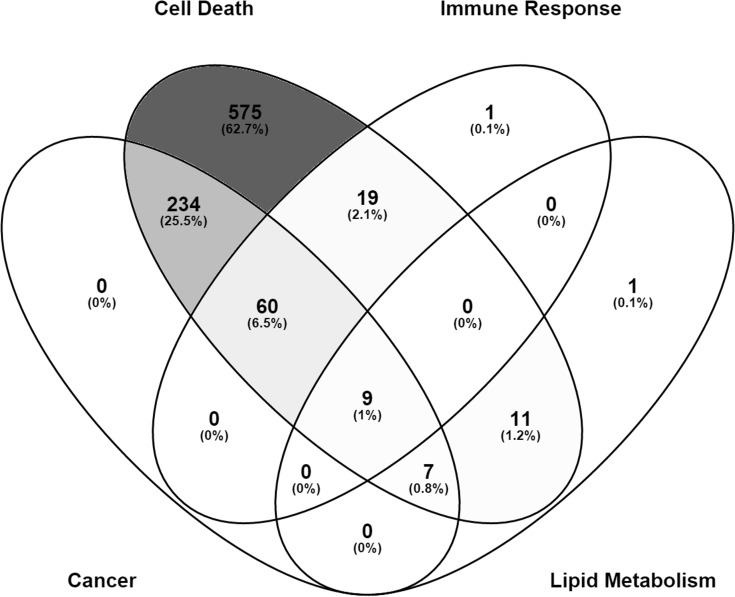
Gene expression patterns affected by IL-15 *in vivo*. Microarray analysis was performed with murine tumor samples. IL-15 treatment affected the expression of 917 genes in total. These were grouped into 4 top diseases and functions: cancer, cell death, immune response, and lipid metabolism. Out of these, 575 were solely associated with Cell death, 234 were associated to cancer and cell death, and 60 were associated cancer, cell death, and immune response. Image was generated using Venny 2.1 [27]

To identify the effect of IL-15 in tumor biology at the genomic level, we performed microarray analysis with RNA extracted from frozen tumor samples. IL-15 treatment deregulated the expression of 917 genes classified in 4 broad diseases and functions: cancer, cell death, immune response and lipid metabolism. Interestingly, most of the genes associated with cancer, were also associated with cell death, for a total of 234 genes (Fig 10). This suggests that IL-15 treatment could promote death of cancer cells. Additionally, 60 genes were associated with cancer, cell death and immune response. Out of the 28 genes associated with lipid metabolism, 9 were associated with cell death, cancer and immune response (Fig 10). These data suggest that IL-15 activity can promote and inflammatory response that results in the death of cancer cells.

**Fig 11:**
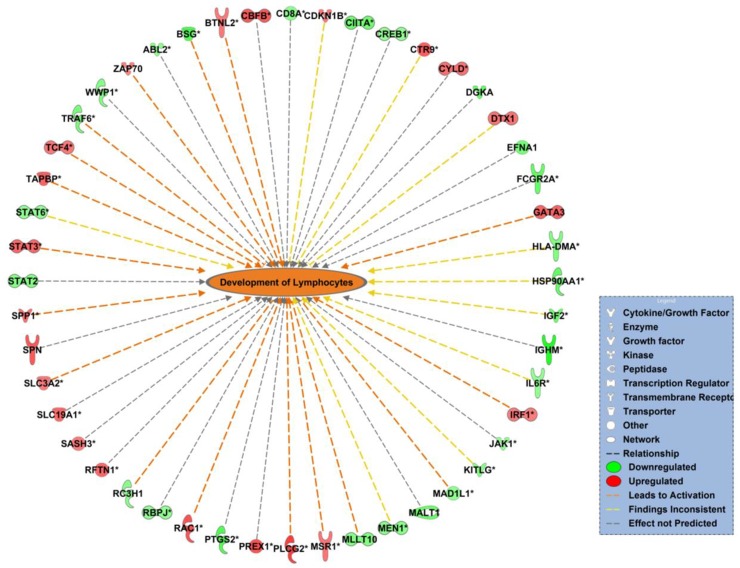
IL-15 affects genes associated with lymphocyte development in a PCa murine model. Network representation of affected functions by IL-15. Orange color represents predicted activation of the network. Orange arrows represent that the state of expression of the gene leads to activation of the network, yellow arrows represent that the state of expression of that gene results in inconclusive activation of the network, and grey arrows represent that the state of expression of that gene does not affect the activation of the network. Red-colored genes are up-regulated in the data set and green-colored genes are down-regulated in the data set. Image was generated in IPA software. p value = 0.016, z score = 1.496

**Fig 12:**
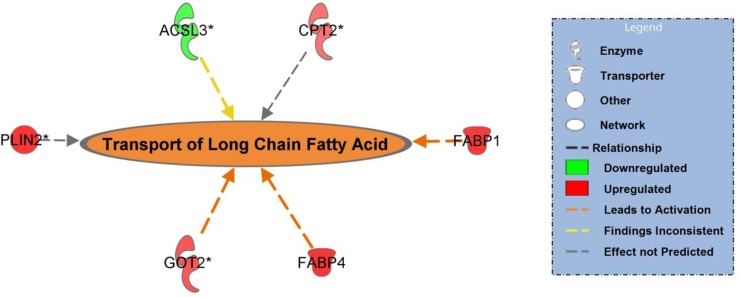
IL-15 affects genes associated with long chain fatty acid transport in a PCa murine model. Network representation of long chain fatty acid transport. This function is represented with an orange color since it was predicted to be increased by IL-15. Up-regulated genes are represented by a red color and down-regulated genes are represented by a green color. Genes whose expression lead to activation of the network are connected with orange arrows, those with an inconclusive connection to the network, are connected with yellow arrows. Genes that do not affect the activation of the network are connected with grey arrows. Image was generated in Ingenuity Pathways Analysis (IPA) software. p value = 0.0031, z score = 1.227

To validate the diseases and functions affected by IL-15, we performed real time PCR assays. Since we reported changes in inflammatory response and lipid metabolism *in vivo*, we chose to confirm these networks. For the immune response network, one relevant function was development of lymphocytes (Fig 11). We selected the following genes from this function: Phospholipase C, Gamma 2 (PLCG2), Ras-Related C3 Botulinum Toxin Substrate 1 (RAC1), Paf1/RNA Polymerase II Complex Component (CTR9), TAP Binding Protein (TAPBP), GATA Binding Protein 3 (GATA3), Signal Transducer and Activator of Transcription 3 (STAT3), Deltex 1, E3 Ubiquitin Ligase (DTX1), Macrophage Scavenger Receptor 1 (MSR1), and Transcription Factor 4 (TCF4) based on the fold change and concordance with the network (Table 4). Within the lipid metabolism network, one relevant function was long chain fatty acid transport from which we chose these genes: Acyl-CoA Synthetase Long-Chain Family Member 3 (ACSL3), Carnitine Palmitoyltransferase 2 (CPT2), Fatty Acid Binding Protein 1 (FABP1), Fatty Acid Binding Protein 4 (FABP4), Glutamic-Oxaloacetic Transaminase 2 (GOT2), and Perilipin 2 (PLIN2) (Fig 12) (Table 4). The primers used for real time PCR confirmation are listed in Table 5.

**Table 4:** Microarray analysis: Genes associated with immune response and lipid metabolism functions, affected by IL-15 *in vivo*.

**Gene Symbol**	**Accession Number**	**Description**	**Fold Change**	**p value**
PLCG2	NM_002661.4	Phospholipase C, Gamma 2 (Phosphatidylinositol-Specific)	2.24	0.0311
RAC1	NM_006908.4	Ras-Related C3 Botulinum Toxin Substrate 1	2.01	0.0276
CTR9	NM_014633.4	Paf1/RNA Polymerase II Complex Component	1.87	0.0301
TAPBP	NM_172209.2	TAP Binding Protein (Tapasin)	1.82	0.0214
GATA3	NM_001002295.1	GATA Binding Protein 3	1.81	0.0142
STAT3	NM_213662.1	Signal Transducer and Activator of Transcription 3	1.75	0.0122
DTX1	NM_004416.2	Deltex 1, E3 Ubiquitin Ligase	1.63	0.0257
MSR1	NM_138716.2	Macrophage Scavenger Receptor 1	1.62	0.0311
TCF4	NM_001306208.1	Transcription Factor 4	1.58	0.0273
ACSL3	NM_004457.3	Acyl-CoA Synthetase Long-Chain Family Member 3	-2.430	0.0137
CPT2	NM_000098.2	Carnitine Palmitoyltransferase 2	1.630	0.0496
FABP1	NM_001443.2	Fatty Acid Binding Protein 1	2.320	0.0184
FABP4	NM_001442.2	Fatty Acid Binding Protein 4	2.910	0.0321
GOT2	NM_002080.3	Glutamic-Oxaloacetic Transaminase 2	1.970	0.0227
PLIN2	NM_001122.3	Perilipin 2	2.950	0.0105

**Table 5:** Microarray analysis: Primer sequences for qRTPCR confirmation of genes affected by IL-15 *in vivo*.

**Gene Symbol**	**Accession Number**	**Forward Primer**	**Reverse Primer**
PLCG2	NM_002661.4	ATGGAGGATGAGCTGGAAATG	GCTTCTCTTTGGGCCCTTAT
RAC1	NM_006908.4	CCTGTAGTCGCTTTGCCTATT	CTCGCCAGTGAGTTAAGTTGTA
CTR9	NM_014633.4	GATGACACTGATGATGACCTACC	CCTCCTCATCTTCACCTTCTTG
TAPBP	NM_172209.2	TCCCAAAGTGCTGGGATTAC	AGAGCTCCAAGAAGGTGAATG
GATA3	NM_001002295.1	GGCGCCGTCTTGATACTT	TCCGTCTCTCTCTCTTCTTCTC
STAT3	NM_213662.1	AGGGTACATCATGGGCTTTATC	CTCCTTCTTTGCTGCTTTCAC
DTX1	NM_004416.2	CCTAGTTTGGGCCGATGTATT	TGGTTGGCCGAAGACAATTA
MSR1	NM_138716.2	TTTGCTTCCTCCGAATCCTAAA	CCAATGAGAGGGATGAGAACTG
TCF4	NM_001306208.1	CTCTCTCCTCCTCCCTGAATAA	TGAGTGCAGAATGTACCACTAAA
ACSL3	NM_004457.3	ATCTGTTTCTGCTGTCCTGTT	CCACTCTGCCAGTATTGTAGTC
CPT2	NM_000098.2	CCTGCATACGGGCAGATAAA	ACACCAAAGCCATCAGAGAC
FABP1	NM_001443.2	CTGGGTCCAAAGTGATCCAA	TGTCACCTTCCAACTGAACC
FABP4	NM_001442.2	CGTCACTTCCACGAGAGTTTAT	TCCCACAGAATGTTGTAGAGTTC
GOT2	NM_002080.3	GGCTTATATGGTGAGCGTGTAG	GGAATACATGGGACGGATCAAG
PLIN2	NM_001122.3	GATTGAGGAGAGACTGCCTATTC	CAGTAGTCGTCACAGCATCTT

**Fig 13:**
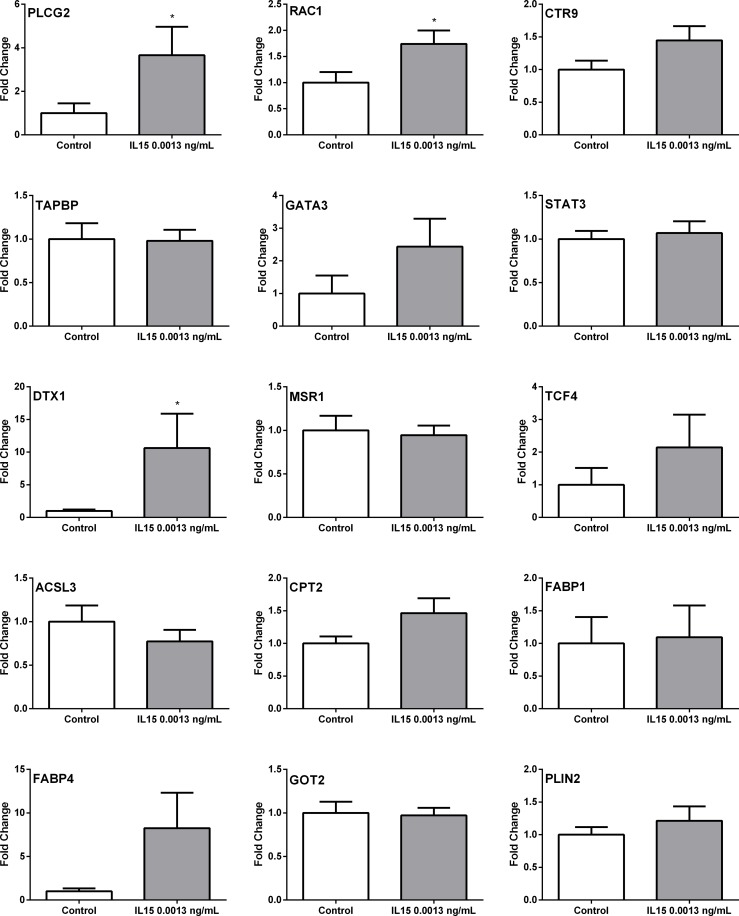
Real time PCR analysis of differentially expressed genes in tumors treated with IL-15. Real time PCR results for: Phospholipase C, Gamma 2 (PLCG2), Ras-Related C3 Botulinum Toxin Substrate 1 (RAC1), Paf1/RNA Polymerase II Complex Component (CTR9), TAP Binding Protein (TAPBP), GATA Binding Protein 3 (GATA3), Signal Transducer and Activator of Transcription 3 (STAT3), Deltex 1, E3 Ubiquitin Ligase (DTX1), Macrophage Scavenger Receptor 1 (MSR1), Transcription Factor 4 (TCF4), Acyl-CoA Synthetase Long-Chain Family Member 3 (ACSL3), Carnitine Palmitoyltransferase 2 (CPT2), Fatty Acid Binding Protein 1 (FABP1), Fatty Acid Binding Protein 4 (FABP4), Glutamic-Oxaloacetic Transaminase 2 (GOT2), and Perilipin 2 (PLIN2). Fold change calculated with the ddCT method. N = 5 representative tumor samples per treatment. Mean+ SEM. *p<0.05. Experiments performed in duplicate.

Our real time PCR results for the genes associated with lymphocyte development show that, IL-15 causes a significant increase in expression of PLCG2, RAC1, GATA3, and DTX1 (Fig 13). In addition, although not statistically significant, IL15 caused an increase in expression of CTR9 and TCF4. The genes TAPBP, STAT3, and MSR1 showed no significant differences. For genes associated with lipid metabolism, real time PCR results showed that IL-15 causes a significant increase of CPT2 mRNA. Although not statistically significant (P>0.05) we identified a notable increase of FABP4 and PLIN2, a visible decrease of ACSL3 mRNA. FABP1 and GOT2 mRNA expression was not visibly different when compared to the control. The fold change results for the real time PCR assays are in Table 6.

**Table 6:** qRT-PCR confirmation of genes associated with immune response and lipid metabolism functions, affected by IL-15 *in vivo*.

**Gene Symbol**	**Description**	**Fold Change**	**p value**
PLCG2	Phospholipase C, Gamma 2 (Phosphatidylinositol-Specific)	3.66	0.035
RAC1	Ras-Related C3 Botulinum Toxin Substrate 1	1.74	0.005
CTR9	Paf1/RNA Polymerase II Complex Component	1.45	0.052
TAPBP	TAP Binding Protein (Tapasin	0.98	0.446
GATA3	GATA Binding Protein 3	2.43	0.015
STAT3	Signal Transducer And Activator Of Transcription 3 (Acute-Phase Response Factor)	1.07	0.335
DTX1	Deltex 1, E3 Ubiquitin Ligase	10.62	0.031
MSR1	Macrophage Scavenger Receptor 1	0.95	0.441
TCF4	Transcription Factor 4	2.15	0.295
ACSL3	Acyl-CoA Synthetase Long-Chain Family Member 3	0.77	0.322
CPT2	Carnitine Palmitoyltransferase 2	1.46	0.044
FABP1	Fatty Acid Binding Protein 1	1.09	0.450
FABP4	Fatty Acid Binding Protein 4	8.26	0.362
GOT2	Glutamic-Oxaloacetic Transaminase 2	0.97	0.446
PLIN2	Perilipin 2	1.21	0.323

## Discussion

Previous work established that IL-15 over-expression is associated with recurrence-free survival [17]. Given its role in the development of NK and cytotoxic T cells, IL-15 has been identified as an anti-tumor cytokine in models for breast and colorectal cancer [14–16,28]. Although these data suggest that IL-15 expression may provide a benefit for PCa patients, the precise role of IL-15 in PCa progression is largely unknown. In this work, we evaluated the effects of IL-15 in PCa using *in vitro* and *in vivo* models. We focused on cell migration, invasion, tumor growth, proliferation, angiogenesis, inflammation and changes in gene expression.

The data described demonstrate that IL-15 has an effect on PCa cells regardless of androgen sensitivity. Effects were significant in PC3 cells which are androgen insensitive and 22Rv1 cells which are androgen sensitive. We observed that when cells were treated with IL-15, migration and invasion were decreased by 30% and 50% respectively. In addition, we confirmed that cell proliferation was not affected (data not shown). These data suggest that the effect we observed is specifically associated with motility and it is not affected by changes in cell growth *in vitro*. Although our *in vivo* data shows an increase in tumor size, proliferation markers such as pH3, were decreased contrasting with our data *in vitro* in which growth remained unchanged. This suggests that other mechanisms caused an increase in tumor volume but inhibited PCa cell proliferation. Interestingly, we determined that IL-15 caused an increased influx of inflammatory cells to the tumor site, and an increase in adipocytes. Which caused an increase in size. In addition to proliferation we also studied metastatic potential. To do so, we evaluated desmin and a-sma expression. Unexpectedly we observed that the expression of both mesenchymal markers was increased. In addition to promoting a mesenchymal phenotype, these proteins are also stromal markers. Although the prostate stroma is mostly of muscle origin; it can become reactive as PCa progresses [29]. Reactive stroma is composed of myofibroblasts and fibroblasts stimulated to express extracellular matrix components. In comparison with the normal prostate stroma, reactive stroma tends to lose the muscle component [30,31]. Studies have characterized the different marker expression patterns that identify a reactive stroma and reports show that an increased a-sma and desmin expression in such stroma correlates with recurrence-free survival [30,31]. This suggests that IL-15 can cause changes in the stroma that promote recurrence-free survival.

Given the role of IL-15 in inflammation, we looked at the infiltration of neutrophils. Neutrophils are involved in the innate immune response, which we were able to assess in our mouse model [32]. The effects of IL-15 in neutrophil function have been studied and results show that IL-15 increases neutrophil invasion, promotes degranulation and increases IL-8 secretion [33]. IL-8 is a pro-inflammatory cytokine that also promotes neutrophil recruitment [34]. On the other hand, neutrophils are mostly unaffected by the similar cytokine, IL-2, even though they express the IL-2 receptor [33]. Therefore, neutrophils infiltration is an appropriate measure of inflammation in our model. Interestingly, we observed an increased amount of neutrophil infiltration in the tumor tissue when compared to the control. Moreover, we were able to verify these data with an increase in neutrophil elastase expression. Neutrophil elastase is a granulocyte-derived serine protease with a major role in host defense against microbes [35]. The fact that secreted serine proteases can regulate inflammation by increasing neutrophil infiltration has implications in tissue injury by chronic or persistent inflammation [36]. Together, these data suggest that IL-15 increased inflammation and promoted neutrophil infiltration as well as degranulation. Nevertheless, other proteases and factors are contained in the granules of neutrophils, which can also contribute to the modulation of inflammatory processes. This information can give further insight to the degree of inflammation and subsequent biological processes that affect PCa progression [36,37].

As previously mentioned, gross examination revealed that, the texture and tissue integrity of IL-15 tumors was considerably softer than control tumors. With this in mind we decided to evaluate the presence of adipocytes and lipid droplets. As predicted, we observed an increased amount of lipid droplets as well as adipocytes increased in size. We then decided to look at the expression of cytokines released by adipocytes and we were able to observe an increase in adiponectin expression. Adiponectin is a signaling molecule, also known as an adipocytokine or adipokine, which is released by adipose tissue. Although one of the functions of adiponectin is the stimulation of lipid metabolism, it is also associated with inflammation [38,39]. In fact, adipose tissue is considered an endocrine organ capable of secreting numerous signaling molecules and promoting inflammation. Moreover, obesity and other metabolic conditions are risk factors for autoimmune diseases, chronic inflammation, and cancer [39,40]. Previous studies suggest that tumor growth can be affected by the increase in lipids. Even though adiponectin signals for lipid metabolism, previous studies have shown that lipid content does not decrease from differentiated adipocytes [41]. This fact supports our data, as we also observed an increased amount of adipocytes in our tissue samples. The role of adiponectin in cancer has been long disputed, in some models it has been linked to progress cancer, while in others it has been identified as a tumor suppressor. This disparity often relies on the cell type and the tumor microenvironment [42,43]. Nevertheless, previous studies have shown that adiponectin inhibits PCa cell growth and that lower concentrations of adiponectin are inversely correlated with PCa malignancy [44,45]. These studies support our findings, as we observed decreased expression of proliferation markers.

Using the PCR array method and real time PCR, we were able to confirm the increased expression of TIMP2. In addition, we confirmed an increase in PTEN, IRS1, IGF1, PIK3CG, FASLG, ILK, CD14, and CCND1 mRNA expression. Up to this point, the data we have gathered show that *in vitro* IL-15 decreases motility of PCa *in vitro* and increases tumor volume, inflammation and neutrophil mobility *in vivo*. Even though MMPs are highly associated with progressive cancer, they are also modulators of inflammatory processes [46]. The active secretion of neutrophil elastase can increase levels of MMPs [47]. To our surprise, TIMPs, natural MMP inhibitors, were also increased in our studies suggesting ECM remodeling. In addition, the increased expression of CD14, an important molecule for the modulation of the innate immune system, and FASLG are indicatives of inflammation [48]. Previous studies show that FASLG stimulates neutrophil infiltration, which supports our findings [49]. Additionally, the increased expression of ILK may suggest an inflammatory phenotype due to its implication in inflammatory cell mobilization[50]. The increase of PTEN, a tumor suppressor, shows that IL-15 may induce an anti-tumor phenotype. However, the increased expression of CCND1, IRS1 and IGF1 which are implicated in cell cycle progression suggest otherwise [51,52].

To further investigate the gene expression patterns modulated by IL-15 we validated the expression of genes associated with lymphocyte development and lipid metabolism. Our results confirmed that IL-15 causes a significant increase in expression of PLCG2, RAC1, GATA3 and DTX1, which are associated with lymphocyte development. The increased expression of PLCG2 suggests that there is increased signaling by IL-15, thereby promoting immunity [32]. Phospholipase c gamma 2 plays an important role in lymphocyte selection during maturation as it promotes T cell receptor signal transduction [53]. In addition, PLCG2 is important for innate immunity because of its role promoting NK cell development and cytotoxicity [54]. The increased expression of RAC 1, a Rho GTPase, has been long associated with cancer progression and cell survival, reason why it is a potential therapeutic target [55,56]. However RAC has also been shown to increase immune response by promoting NK and CD8+ T cells cytotoxicity [57]. The up-regulation of GATA3 has been also associated with cancers some being of hematopoietic origin [58]. GATA3 is a transcription factor involved in the development of lymphocytes, particularly favoring a Th2 response [59,60]. Increased expression of GATA3 has been shown to be a good prognosis marker in other types of cancer such as breast. However, mutations in this gene have shown opposite outcomes [61]. DTX1 on the other hand, functions as an ubiquitin binding protein and has been shown to function in B cell maturation [62]. However, over-expression of this protein can result in T cell anergy and can promote cancer proliferation [62,63]. The confirmation of these genes suggests that IL-15 promotes inflammation. Nonetheless, more studies can be done to ensure that this expression results in an antitumor response given that some of these genes can also be associated with cancer progression.

## Conclusion

PCa is often treated with radical prostatectomy (RP) with adjuvant therapy like radiation or chemotherapy. Even though a combinatorial treatment can provide positive results, 15–30% of the patients will suffer from biochemical recurrence or elevated blood levels of PSA [2,3]. Although the early detection of potential metastatic or recurrent PCa can lead to proactive use of adjuvant therapeutic options, the available biomarkers and (or) clinical information are insufficient to predict recurrence and metastasis [7].

Since inflammatory processes play a significant role in cancer progression, the study of inflammatory mediators, such as cytokines, is important for PCa. Reason why, cytokines have been studied as potential biomarkers for progression and recurrence. Previous work established that IL-15 over expression was associated with recurrence-free survival suggesting that IL-15 could be a potential biomarker [17]. In this study, we evaluated several hallmarks of cancer such as: proliferation, cell motility, tumor growth, angiogenesis, inflammation and changes at the genetic level. Our data show that IL-15 decreases cell migration and invasion *in vitro*. Additionally, the presence of IL-15 in the tumor microenvironment, decreases proliferation, and blood vessel formation. We also observed that IL-15 increases tumor volume as a consequence of inflammation and lipid mobilization.Further *in vivo* studies are needed to have a better understanding given that inflammation, neutrophil infiltration and obesity are risk factors. Nevertheless, we show that IL-15 affects PCa by decreasing motility affecting inflammatory processes, and modifying gene expression. All these factors are relevant for PCa progression and should be taken into consideration while evaluating IL-15 as a biomarker and for further applications in the clinic.

## Supporting information

S1 Fig Control a sample hybridization scan. Image file generated after hybridization process of a control sample.
(JPG)

S2 Fig Control b sample hybridization scan. Image file generated after hybridization process of a control sample.
(JPG)

S3 Fig IL-15 a sample hybridization scan. Image file generated after hybridization process of an IL-15 sample.
(JPG)

S4 Fig IL-15 b sample hybridization scan. Image file generated after hybridization process of an IL-15.
(JPG)

S5 Fig Relative probe cell intensity. Relative intensity box plot per sample.
(PNG)

S6 Fig Signal histogram. Representative image of the intensity histogram per sample.
(PNG)

S1 File Supporting information. Detailed information pertaining the microarray experiments.
(DOCX)

S2 File HuGENE probe sequences. The Probe Sequence files for the HuGene 2.0 array chip in FASTA format.
(ZIP)

S3 File Control a hybridization scan raw data. Raw data obtained from hybridization scan corresponds to control sample a.
(CEL)

S4 File Control hybridization scan raw data. Raw data obtained from hybridization scan corresponds to a control sample b.
(CEL)

S5 File IL-15 hybridization scan raw data. Raw data obtained from hybridization scan corresponds to an IL-15 sample a.
(CEL)

S6 File IL-15 hybridization scan raw data. Raw data obtained from hybridization scan corresponds to an IL-15 sample b.
(CEL)

S7 File Control a normalized expression data. Normalized data obtained after Gene Level RMA sketch analysis with the Expression Console Software (Affymetrix Inc, Santa Clara, CA)
(CHP)

S8 File Control b normalized expression data. Normalized data obtained after Gene Level RMA sketch analysis with the Expression Console Software (Affymetrix Inc, Santa Clara, CA)
(CHP)

S9 File IL-15 a normalized expression data. Normalized data obtained after Gene Level RMA sketch analysis with the Expression Console Software (Affymetrix Inc, Santa Clara, CA)
(CHP)

S10 File IL-15 b normalized expression data. Normalized data obtained after Gene Level RMA sketch analysis with the Expression Console Software (Affymetrix Inc, Santa Clara, CA)
(CHP)

S11 File QC metrics summary. Tabular representation of all QC metrics.
(TXT)

S12 File Gene expression summary. Gene level expression analysis results.
(TXT)
