# Supplementary figures and images for: IL-15 regulates migration, invasion, angiogenesis and genes associated with lipid metabolism and inflammation in prostate cancer
Source: PLoS One. 2017 Apr 5;12(4):e0172786. doi: 10.1371/journal.pone.0172786 (PMC5381801; doi:10.1371/journal.pone.0172786)

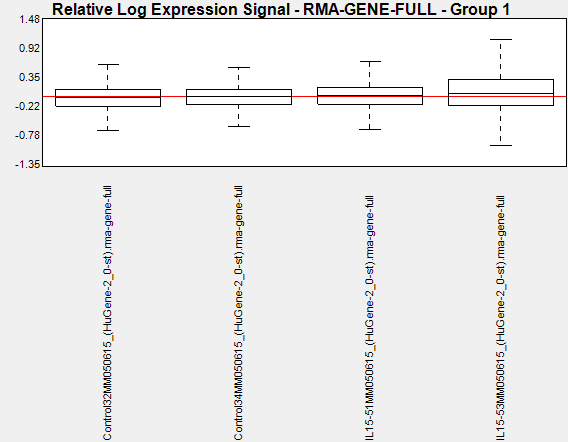

Supplement: S5 Fig — Relative intensity box plot per sample. (PNG) [file pone.0172786.s005.PNG]

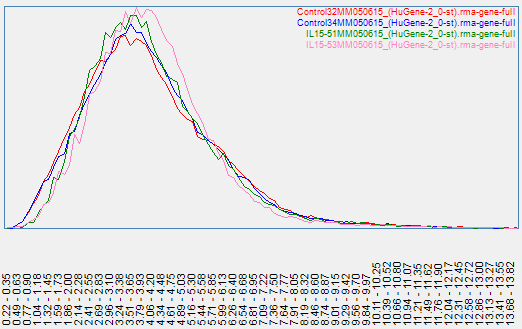

Supplement: S6 Fig — Representative image of the intensity histogram per sample. (PNG) [file pone.0172786.s006.PNG]
