# Supplementary material for: IL-15 regulates migration, invasion, angiogenesis and genes associated with lipid metabolism and inflammation in prostate cancer
Source: PLoS One. 2017 Apr 5;12(4):e0172786. doi: 10.1371/journal.pone.0172786 (PMC5381801; doi:10.1371/journal.pone.0172786)
Supplement: S1 File — Detailed information pertaining the microarray experiments. (DOCX) [file pone.0172786.s007.docx]

# IL-15 regulates migration, invasion, angiogenesis and genes associated with lipid metabolism and inflammation in prostate cancer.

**Authors:** Krizia Rohena-Rivera^1,2^, María M. Sánchez-Vázquez^2^, Diana A. Aponte-Colón^2^, Ingrid S. Forestier-Román^1,2^, Mario E. Quintero-Aguiló^2^, Magaly Martínez-Ferrer^1,2,3^

**Supplemmentary information:**

**Microarray analysis**

**Experimental Design**

The purpose of the study was to determine the differences in gene expression between murine tumors generated under two conditions, Vehicle and IL-15 0.0013 ng/mL. For this procedure, we used an orthotopic xenograft model. In this case, 250,000 22RV1 cells were injected in the anterior prostate lobes of ICR-SCID mice. The treatment, IL-15 (0.0013 ng/mL) or vehicle (Saline) was administered bi-weekly with intraperitoneal injections during 4 weeks. In total, the control group had 13 mice and the IL-15 group had 10 mice. Each mouse yielded 2 tumors from which one was snap frozen for RNA extraction and analysis.

**Method Summary**

Affymetrix gene chip based transcript profiling was performed at the RCMI Center for Genomics in Health disparities and Rare Diseases (University of Puerto Rico, Medical Sciences Campus). Following quality control, the RNA was prepared for microarray analysis using the standard Affymetrix protocol (Affymetrix Inc, Santa Clara, CA). Total RNA (100 ng) was converted to cDNA and amplified using T7 oligo dT and the GeneChip® WT cDNA Synthesis Kit, the GeneChip® WT cDNA Amplification Kit, and the GeneChip® Sample Cleanup Module as described in the GeneChip® Whole Transcript (WT) Sense Target Labeling Assay Manual Addendum. All quality control steps were followed to ensure that the RNA was adequate for later use in the first strand cDNA synthesis (where 10 μg are required), that the yield of cDNA was ≥ 5.5 µg of Single-Stranded DNA and that the fragmentation step worked properly by size analysis with the RNA 6000 Nano LabChip Kit in the Agilent Bioanalyzer. A gel-shift analysis of the WT (Whole Transcript) was done to assess the labeling efficiency of the fragmented cDNAs. The image data was normalized using the Expression Console software provided by Affymetrix. The mode of analysis used was Gene Level RMA sketch. The QC metrics were verified to certify that the hybridization was performed correctly. The signal distribution among arrays was observed to certify that all arrays exhibit a uniform signal distribution. The intensity boxplot was observed to ensure that all samples had uniform intensity values to proceed with the analysis. Gene expression values and clustering was done using the Transcriptome Analysis Console also provided by Affymetrix. The settings used to identify differences in expression were, a fold change higher that 2 or lower than -2 and a p value lower than 0.05. Identification of gene expression patterns was done with IPA software. The settings for this final analysis were a fold change higher than 1.5 or lower than -1.5 and a p value lower than 0.05. To identify the affected functions and networks we used the “diseases and functions” sections of the IPA software. We selected the complete array of functions and selected the top four representative functions based on score.

**Microarray Chip**

Affymetrix GeneChip® Human Gene 2.0 ST Array (Cat No. 902459)

More information available at <http://www.affymetrix.com/catalog/131453/AFFY/Human+Gene+ST+Arrays#1_3>

**Software**

Expression Console. Available from Affymetrix

<http://www.affymetrix.com/estore/catalog/131414/AFFY/Expression+Console+Software>

Transcriptome Analysis Console. Available from Affymetrix

<http://www.affymetrix.com/estore/catalog/prod760001/AFFY/Transcriptome+Analysis+Console+%28TAC%29+Software>

**Array Design:**

The array used for this experiment was Affymetrix GeneChip® Human Gene 2.0 ST Array. We used the Affymetrix Gene arrays because they offer whole-transcript coverage. This microarray chip consists of a square glass slide encased in a plastic cartridge. The oligonucleotide probes are mounted on the glass slide which remains protected in a chamber within the plastic cartridge. Before use, these cartridges were stored at 4°C as indicated by the manufacturer. More information about the array format and the specifications can be found at:

<http://www.affymetrix.com/catalog/131453/AFFY/Human+Gene+ST+Arrays#1_3>

<http://media.affymetrix.com/support/downloads/package_inserts/703132_3%20Generic%20Gene%20WT%20100-Fmt%20Array%20PI.pdf>

<http://www.affymetrix.com/support/technical/datasheets/hugene_2_st_datasheet.pdf>

**S2 File. HuGENE Probe Sequences.**  The Probe Sequence files for the HuGene 2.0 array chip in FASTA format.

**Sample Preparation**

RNA was extracted from snap frozen tumor samples using the RNeasy Mini Kit (Qiagen Inc., Valencia; CA, USA) following the directions of the manufacturer with some modifications. These tumors were generated with 22RV1 cells in ICR SCID mice over a period of 4 weeks. Briefly, a 30ug section of the tumor was macerated in dry ice, subsequently transferred to 350 µL of lysis buffer. Final homogenization was done over ice with a rotor stator homogenizer in 5 second intervals. To increase RNA yield, the tissue lysate was treated with Proteinase K (Qiagen Inc., Valencia; CA, USA) for 10 minutes at 55 °C. Afterwards, the debris was separated by centrifugation and the cleared lysate was used to continue the RNA extraction following the instructions. To increase RNA purity, we performed on column DNase digestion (Qiagen Inc., Valencia; CA, USA). Extracted RNA was stored at -80 °C. These samples where then transferred to the RCMI Center for Genomics in Health disparities and Rare Diseases (University of Puerto Rico, Medical Sciences Campus) where the quality of the RNA was assessed and the microarray procedure was performed.

**Hybridization**

After measuring RNA concentration, 100 ng of RNA was converted to cDNA and amplified using T7 oligo dT and the GeneChip® WT cDNA Synthesis Kit, the GeneChip® WT cDNA Amplification Kit and the GeneChip® Sample Cleanup Module exactly as described in the GeneChip® Whole Transcript (Wt) Sense Target Labeling Assay Manual Addendum. All quality control steps were followed to ensure that the synthesis was adequate for later use. It was confirmed that the yield of cDNA was ≥ 5.5 µg of Single-Stranded DNA and that the fragmentation step worked properly by size analysis with the RNA Nano Assay 6000 in an Agilent Bioanalyzer. All microarray hybridizations were carried out simultaneously to minimize batch variation. Double-stranded cDNA synthesis, in vitro transcription, Synthesis of first-strand DNA, fragmentation, and labeling, hybridization/control mixture preparation, Affymetrix GeneChip® Human Gene 2.0 ST Array hybridization, and post-processing procedures were performed at the RCMI Molecular Genetics Core Facility of the UPR MSC exactly as recommended by the manufacturer. Hybridization cocktail was prepared as directed by the manufacturer making sure to include the eukaryotic hybridization controls BioB, BioC, BioD and Cre. These controls are then used to measure the efficiency of the procedure. Array hybridization was done for 16 hours at 45 °C in a hybridization oven with 60 rpm rotation. Following washing procedures were done using the automated fluidics station system as required by the manufacturer.

**Measurements**

Hybridization scan images can be viewed in JPG format.

**S1 Fig. Control Sample Hybridization Scan.** Image file generated after hybridization process of a control sample.

**S2 Fig. Control Sample Hybridization Scan** Image file generated after hybridization process of a control sample.

**S3 Fig. IL-15 Sample Hybridization Scan** Image file generated after hybridization process of an IL-15 sample.

**S4 Fig. IL-15 Sample Hybridization Scan** Image file generated after hybridization process of an IL-15.

Raw data obtained from hybridization process are attached separately in CEL format.

**S3 File. Control Hybridization Scan Raw Data.** Raw data obtained from hybridization scan corresponds to a control sample

**S4 File. Control Hybridization Scan Raw Data.** Raw data obtained from hybridization scan corresponds to a control sample

**S5 File. IL-15 Hybridization Scan Raw Data.** Raw data obtained from hybridization scan corresponds to an IL-15 sample

**S6 File. IL-15 Hybridization Scan Raw Data.** Raw data obtained from hybridization scan corresponds to an IL-15 sample

Normalized data obtained after Gene Level RMA sketch analysis with the Expression Console Software are attached in CHP format.

**S7 File. Control Normalized Expression Data.** Normalized data obtained after Gene Level RMA sketch analysis with the Expression Console Software (Affymetrix Inc, Santa Clara, CA)

**S8 File. Control Normalized Expression Data.** Normalized data obtained after Gene Level RMA sketch analysis with the Expression Console Software (Affymetrix Inc, Santa Clara, CA)

**S9 File. IL-15 Normalized Expression Data.** Normalized data obtained after Gene Level RMA sketch analysis with the Expression Console Software (Affymetrix Inc, Santa Clara, CA)

**S10 File. IL-15 Normalized Expression Data.** Normalized data obtained after Gene Level RMA sketch analysis with the Expression Console Software (Affymetrix Inc, Santa Clara, CA)

**Normalization and Analysis**

For analysis, we obtained CEL files from the RCMI Center for Genomics in Health disparities and Rare Diseases (University of Puerto Rico, Medical Sciences Campus). These files were imported as a new study in the Expression Console software. Afterwards, we performed Gene Level RMA sketch analysis from which we generated CHP files (see attached). These files were then subjected to QC metrics within the same software. We observed the relative probe cell intensity as well as the signal histogram to identify any outliers in our data set.

**S5 Fig. Relative Probe cell intensity.** Relative intensity box plot per sample.

**S6 Fig. Signal Histogram.** Representative image of the intensity histogram per sample.

To verify if hybridization was efficient we observed the spike controls

AFFX-r2-Ec-BioB

AFFX-r2-Ec-BioC

AFFX-r2-Ec-BioD

AFFX-r2-P1-Cre

We also observed if sample labeling was done properly by observing the internal labeling controls Briefly, eukaryotic GeneChip probe array contains probe sets from several *B. subtilis* genes that are absent in eukaryotic samples (*lys*, *phe*, *thr*, and *dap*). These polyadenylated transcripts premixed at staggered concentrations

Lys: AFFX-r2-Bs-lys (1:100,000)

Phe: AFFX-r2-Bs-phe (1:50,000)

Thr: AFFX-r2-Bs-thr (1:25,000)

Dap: AFFX-r2-Bs-dap (1:6,667)

We made sure that all of the Poly-A controls were present in our samples.

**S11 File. QC Metrics Summary.** Tabular representation of all QC metrics.

After the samples were identified as compliant with QC parameters, CHP files were imported in the Transcriptome Analysis Console software. Within this software we performed a “gene level differential expression analysis” with an unpaired ANOVA at a 95% confidence interval. To identify gene expression patterns, the unfiltered data was analyzed using the IPA. A fold change higher than 1.5 or lower than -1.5 and a p value lower than 0.05 was considered relevant for our study.

**S12 File. Gene Expression Summary.** Gene level expression analysis results.
